# Expression of a hindlimb-determining factor *Pitx1* in the forelimb of the lizard *Pogona vitticeps* during morphogenesis

**DOI:** 10.1098/rsob.160252

**Published:** 2016-10-26

**Authors:** Jane Melville, Sumitha Hunjan, Felicity McLean, Georgia Mantziou, Katja Boysen, Laura J. Parry

**Affiliations:** 1 Museum Victoria, Carlton, Victoria 3001, Australia; 2 School of Biosciences, University of Melbourne, Parkville, Victoria 3010, Australia

**Keywords:** *GHR*, limb development, lizards, *pitx1*, *shh*, squamates

## Abstract

With over 9000 species, squamates, which include lizards and snakes, are the largest group of reptiles and second-largest order of vertebrates, spanning a vast array of appendicular skeletal morphology. As such, they provide a promising system for examining developmental and molecular processes underlying limb morphology. Using the central bearded dragon (*Pogona vitticeps*) as the primary study model, we examined limb morphometry throughout embryonic development and characterized the expression of three known developmental genes (*GHR, Pitx1* and *Shh*) from early embryonic stage through to hatchling stage via reverse transcription quantitative polymerase chain reaction (RT-qPCR) and immunohistochemistry (IHC). In this study, all genes were found to be transcribed in both the forelimbs and hindlimbs of *P. vitticeps.* While the highest level of *GHR* expression occurred at the hatchling stage, *Pitx1* and *Shh* expression was greatest earlier during embryogenesis, which coincides with the onset of the differentiation between forelimb and hindlimb length. We compared our finding of *Pitx1* expression—a hindlimb-determining gene—in the forelimbs of *P. vitticeps* to that in a closely related Australian agamid lizard, *Ctenophorus pictus*, where we found *Pitx1* expression to be more highly expressed in the hindlimb compared with the forelimb during early and late morphogenesis—a result consistent with that found across other tetrapods. Expression of *Pitx1* in forelimbs has only rarely been documented, including via *in situ* hybridization in a chicken and a frog. Our findings from both RT-qPCR and IHC indicate that further research across a wider range of tetrapods is needed to more fully understand evolutionary variation in molecular processes underlying limb morphology.

## 1. Background

The integration of the fields of evolution and developmental biology is leading to significant advances in our understanding of the molecular basis of morphological evolution. However, there has been a historical reliance on model organisms in developmental biology, with most studies focusing on relatively few and evolutionarily distant species that are suitable for embryological and genetic manipulation [1], such as mice, chickens, frogs and zebrafish. A case in point is research on the molecular basis of variation in tetrapod limb morphology. Many molecular aspects of limb development have been studied extensively in model tetrapods, mainly chicken and mouse [2]. However, this represents only a small fraction of tetrapod limb diversity and evolution. One group for which very limited research into the developmental and molecular processes underlying limb morphology has been conducted are reptiles. Reptiles form a key evolutionary group in terrestrial vertebrates, both in terms of morphological diversity and the evolution of the tetrapod limb.

Squamates, which include lizards and snakes, are the most speciose group of reptiles, and provide a promising system for examining developmental and molecular processes underlying limb morphology. Limb morphology and especially hindlimb length are known to influence the ecology of many lizard species [3]. Within lizards, the relationship between limb length and ecology is largely a result of increased hindlimb length, which is associated with increased running speed [4–6]. Recent work on *Anolis* lizards has shown that variation in limb length results from changes occurring very early in embryonic development, prior to formation of the cartilaginous long bone anlagen [7]. *Anolis* lizards serve as an emerging model system for the study of limb development and evolution [7,8], because it is a highly speciose genus that has been well studied, both ecologically and morphologically. Additionally, the *Anolis carolinensis* genome was the first squamate genome to be sequenced [9]. However, understanding the genetic mechanisms of limb development across a variety of organisms can help elucidate the evolutionary processes that lead to morphological diversity, which ultimately allows terrestrial vertebrates to occupy a vast array of ecological niches [10]. Thus, expanding limb developmental research to other squamate lineages has the potential to provide a particularly powerful system to investigate the developmental and molecular basis underlying tetrapod limb diversity.

Australian agamid lizards (Agamidae: Amphibolurinae) form an ideal study system to examine developmental and molecular processes underlying limb morphology. They are a speciose lineage (more than 72 species) with a wide diversity in limb morphology, and agamids have been shown to exhibit convergent morphological and ecological evolution with the clade incorporating pleurodont iguanians [11], which includes the *Anolis* lizards. Moreover, the genome of the Australian agamid *Pogona vitticeps* has recently been sequenced [12]. Agamids, in particular *P. vitticeps*, constitute ideal laboratory animals, because they are oviparous with medium to large clutches, with a well-established history of captive breeding, and they are readily commercially available. Consequently, Australian agamids provide an exciting new avenue for research into the developmental and molecular processes underlying the evolution and diversity in tetrapod limb morphology.

We integrated a morphological study of limb development during embryogenesis in *P. vitticeps* with the molecular investigation of gene expression during embryonic limb development. Our study covered stages of limb development from oviposition (egg laying) to 21 days post-hatching, and thus all stages at which variation in limb length may originate. Although variation in limb length occurs very early during embryogenesis in *Anolis* [7], there are four main developmental phases in which variation could occur: limb-bud initiation, limb-bud outgrowth and patterning, morphogenesis (which is the differentiation and development of limb structures) and growth [13]. We sought to characterize limb development during embryogenesis in Australian agamids and to determine at which stage limb length variation occurs. We then undertook the molecular component of this study to investigate gene expression during embryogenesis.

We selected three genes that are known to be involved at different stages of limb development in model organisms, such as mouse and chicken (see the brief review below). We quantified gene transcription in the forelimbs and hindlimbs of *P. vitticeps* for the paired-like homeodomain transcription factor 1 (*Pitx1*), sonic hedgehog (*Shh*) and growth hormone receptor (*GHR*). We hypothesized, based on previous research in vertebrates, that these genes would each show a peak in transcription levels at different phases of embryonic limb development (i.e. *Pitx1* during limb-bud outgrowth and patterning, *SHH* during morphogenesis and *GHR* during the growth phase). We tested this hypothesis using RT-qPCR for each gene in both forelimbs and hindlimbs across nine developmental stages in *P. vitticeps*. We then examined *Pitx1* expression in forelimbs and hindlimbs using immunohistochemistry (IHC). To determine whether the pattern of *Pitx1* expression in *P. vitticeps* is representative of Australian agamid lizards, we investigated *Pitx1* transcription and expression during morphogenesis in a closely related Australian agamid lizard, *Ctenophorus pictus*.

### 1.1. Limb development genes: *Pitx1, Shh* and *GHR*

*Pitx1* is a hindlimb-determining factor expressed early in development [14,15], with *Pitx1* deletion causing loss of skeletal hindlimb structures in mice [14,16]. Ectopic expression of *Pitx1* in the developing forelimb of mice and chicks generates some bone and soft tissue features similar to that of the hindlimb [16–18]. More recent work in mice has shown that *Pitx1*, expressed in the hindlimb bud mesenchyme, is also necessary for normal expression of *Tbx4*, a transcription factor required for normal hindlimb development [19]. *Pitx1* has been found to be enriched on hindlimb *cis-*regulatory elements but is also strongly associated with many functionally verified limb enhancers [20]. These findings have led to the suggestion that *Pitx1* influences hindlimb morphology through the activation of hindlimb-specific enhancers as well as through the hindlimb-specific modulation of enhancers that are active in both sets of limbs [20]. Additionally, in mice, it has been demonstrated that *Pitx1* influences the patterning of different tissue types of the limb [17], including influencing morphogenesis of cartilaginous precursors of bone, the organization of myoblasts into muscle bundles, as well as attachment of tendon cells between bone and muscles. This role in morphogenesis is believed to be complete by stage E14.5 in mice [17], which is the stage at which toes and fingers are clearly separated and the distal-most elements of the fingers have formed.

The only study published on the role of *Pitx1* in limb development of lizards, which used a micromass culture system, found that *Pitx1* transcript levels were maintained in micromasses derived from *A. sagrei* hindlimb cells compared to those in forelimb cells [8]. While the authors found that the core binding sites for PITX1 were not conserved between *Anolis* and mammals, they did detect upregulation of the conserved hindlimb transcription factor *hoxc11* through ectopic expression of PITX1 in *A. sagrei* forelimb cells. These recent results suggest that regulation of *hoxc11* transcription through PITX1 may be mediated through binding sites that are not conserved between lizards and mammals, providing motivation to further explore the role of *Pitx1* in the limb development of squamates.

*Shh* is known to be important in chicken and mouse for limb outgrowth and extension, and determination of digit identity and number. *Shh* is integrally involved in limb development and is essential for maintenance of the apical ectodermal ridge (AER), which is required for limb outgrowth and extension [13]. *Shh* is also required to maintain the zone of polarizing activity (ZPA) [21] which is responsible for anterior–posterior axis formation, thus ensuring that all limb elements are developed in the correct orientation. One of the few studies examining the role of *Shh* in limb development of lizards investigated the skink genus *Hermiergis* [22]*.* Shapiro and co-workers found that changes in the duration of *Shh* expression during early development resulted in variation in the number of digits. While it is assumed that the biological role of limb development genes, such as *Shh*, is highly conserved across species, few studies have looked at limb development genes in non-model species.

*GHR* encodes a transmembrane receptor that binds to growth hormone (GH) and is thought to stimulate growth, cell reproduction and regeneration. It is also involved in regulation of bone growth and is highly conserved across species [23]. Although GH is obligatory for post-natal growth, early embryonic growth has traditionally been viewed as a ‘growth without GH’ syndrome [24]. However, it has been shown that GH may act as an autocrine/paracrine factor during early chick embryogenesis [25]. GH coordinates the growth of multiple target tissues during development, including skeletal muscle [26], where it is highly expressed and induces the synthesis of the insulin-like growth factor I (IGF1) [27]. Both GH and IGF1 are important regulators of longitudinal growth [28,29]. *GHR* has been described as a Z-borne sex chromosome-linked gene in the chicken but an autosomal gene mapped to a contiguous block of chromosome 2 in *P. vitticeps* [30]. To this date, research into *GH* and *GHR* in reptiles is particularly limited, and their role in reptile embryonic limb development remains to be explored.

## 2. Results

### 2.1. Post-oviposition limb development in *Pogona vitticeps*

**Figure 1.**
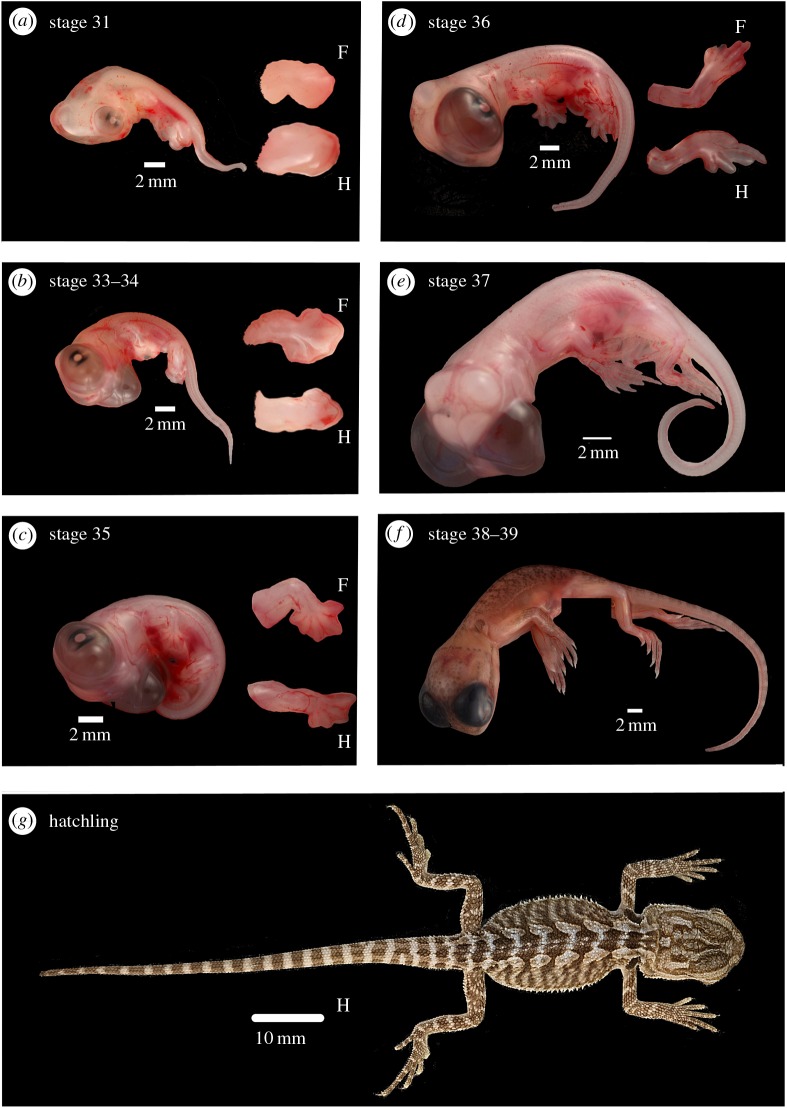
The limb development stages included in our study for *Pogona vitticeps.* Stages include (described in electronic supplementary material, S1): (*a*) stage 31, late limb-bud outgrowth and patterning (9 days post-oviposition); (*b*) stage 33–34, morphogenesis, cartilaginous anlagen of the limb bones form (15 days post-oviposition; qPCR time point T2); (*c*) stage 35, morphogenesis (20 days post-oviposition; qPCR time point T3); (*d*) stage 36, morphogenesis (25 days post-oviposition; qPCR time point T4); (*e*) stage 37, transition from morphogenesis to growth phase (28–29 days post-oviposition; qPCR time point T5); (*f*) stage 38–39, growth, mediated from epiphyseal growth zones (44 days post-oviposition; qPCR time point T6); and (*g*) hatchling (21 days post-hatching; qPCR time point T9). Whole embryos are shown (scales bars provided) and for stages 31–36 enlarged images of forelimb (F) and hindlimb (H) are provided (not to scale).

We determined embryonic limb development stages in *P. vitticeps* according to the developmental stages described for *Lacerta vivipara* [31]. In the following text, the corresponding embryonic stages in *A. sagrei* are also provided [1]. As in *A. sagrei*, early embryogenesis in *P. vitticeps* occurs within the oviducts prior to oviposition, and eggs are laid at the limb-bud outgrowth and patterning stages of development (stage 28–30, *Anolis* stage 3–5). The seven limb development stages included in our study are illustrated in figure 1 and described in electronic supplementary material, S1. The first sampling period included in our study was 9 days post-oviposition, which we determined to be the late limb-bud outgrowth and patterning stage 31 (*Anolis* stage 6). Morphogenesis in *P. vitticeps* occurred 12–29 days post-oviposition, with our sampling period of 28–29 days post-oviposition being defined as late stage morphogenesis, transitioning into the growth phase of limb development (stage 37, *Anolis* stage 12–13). At 44 days post-oviposition, limbs were well developed and in the growth phase of development (stage 38–39, *Anolis* stage 17). Hatching occurred after 59–67 days of incubation at 28°C.

### 2.2. Post-oviposition limb growth in *Pogona vitticeps*

**Figure 2.**
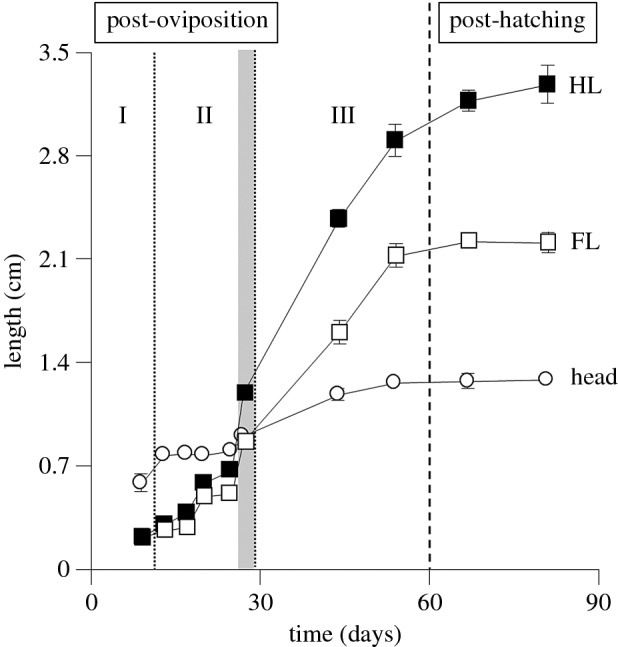
Limb growth during embryogenesis in *Pogona vitticeps.* Mean lengths with standard error bars for each development stage included in the study (as days post-oviposition) are shown for forelimb, hindlimb and head length. Sample size for each development stage can be found in table 1. Each stage of embryogenesis is indicated: I, late limb-bud outgrowth and patterning; II, morphogenesis; III, growth. The shaded vertical bar shows the period of embryogenesis when differentiation between forelimb and hindlimb lengths occurs, with the hindlimb becoming significantly longer than the forelimb.

Our sampling regime in *P. vitticeps* allowed a detailed statistical analysis of embryonic limb growth during embryogenesis. Limb growth was measured across embryonic limb development stages in *P. vitticeps* for the sampling periods included in our study (table 1). A nested ANOVA comparing total length of limb type (forelimb versus hindlimb) within sampling periods showed that there is a significant difference between forelimb and hindlimb lengths across sampling periods (*F*_18,119_ = 108.16, *p* < 0.001). Forelimb and hindlimb lengths were found not to differ significantly during late outgrowth and patterning (day 9 post-oviposition: *F*_1,4_ = 6.84, *p* = 0.06) and both were shorter than head length (figure 2). Differentiation between forelimb and hindlimb lengths occurred during morphogenesis (figure 2), where hindlimb length was found to be significantly longer than forelimb length by the 12–13 d post-oviposition time period (*F*_1,12_ = 7.36, *p* = 0.02). The increasing differentiation of forelimb and hindlimb length continued through the morphogenesis and embryonic growth phases.

**Table 1.** Morphological measurements for the developmental stages of *Pogona vitticeps*. Values presented are mean length (cm) ± s.e. in parentheses. Developmental stages are indicated as days post-oviposition (d) or days post-hatching (dH). Growth rates are provided in per cent per day (% per day), with significant growth rates indicated in italics. Images provided in figure 1.

	9 d	12–13 d	15–18 d	20 d	25 d	28–29 d	44 d	53–55 d	7 dH	21 dH
embryonic staging (figure 1, electronic supplementary material, S1)	31	32	33–34	35	36	37	38–39	40	—	—
qPCR time period (figures 4 and 5)		T1	T2	T3	T4	T5	T6	T7	T8	T9
*N*	3	7	6	6	7	9	6	9	9	7
hindlimb										
proximal	—	—	—	0.22 (0.01)	0.24 (0.00)	0.35 (0.01)	0.71 (0.04)	0.76 (0.04)	0.86 (0.02)	0.87 (0.03)
distal	—	—	—	0.14 (0.01)	0.17 (0.01)	0.30 (0.02)	0.59 (0.02)	0.84 (0.04)	0.95 (0.03)	1.00 (0.03)
autopod	—	—	—	0.21 (0.02)	0.27 (0.04)	0.55 (0.02)	0.39 (0.03)	0.57 (0.03)	0.55 (0.02)	0.56 (0.03)
fourth digit	—	—	—	—	—	—	0.67 (0.02)	0.72 (0.02)	0.81 (0.02)	0.85 (0.04)
total limb length	0.22 (0.00)	0.30 (0.01)	0.38 (0.01)	0.58 (0.02)	0.67 (0.02)	1.19 (0.04)	2.37 (0.06)	2.90 (0.11)	3.17 (0.07)	3.28 (0.13)
growth rate	—	9.09	6.67	17.54	3.10	*38.81*	5.83	2.24	0.72	0.25
forelimb										
proximal	—	—	—	0.18 (0.01)	0.17 (0.00)	0.26 (0.01)	0.52 (0.03)	0.61 (0.03)	0.69 (0.02)	0.67 (0.02)
distal	—	—	—	0.14 (0.01)	0.16 (0.01)	0.22 (0.01)	0.43 (0.03)	0.64 (0.04)	0.66 (0.02)	0.67 (0.03)
autopod	—	—	—	0.17 (0.01)	0.18 (0.01)	0.39 (0.02)	0.23 (0.01)	0.37 (0.01)	0.33 (0.02)	0.32 (0.01)
fourth digit	—	—	—	—	—	—	0.42 (0.03)	0.50 (0.02)	0.54 (0.02)	0.55 (0.03)
total limb length	0.21 (0.00)	0.26 (0.01)	0.28 (0.02)	0.49 (0.02)	0.51 (0.02)	0.86 (0.03)	1.60 (0.08)	2.12 (0.08)	2.22 (0.05)	2.21 (0.07)
growth rate	—	5.95	1.92	25.00	0.82	*34.31*	5.06	3.25	0.36	−0.03
head										
total length	0.58 (0.06)	0.77 (0.02)	0.78 (0.03)	0.77 (0.02)	0.80 (0.03)	0.90 (0.02)	1.18 (0.04)	1.26 (0.02)	1.27 (0.05)	1.28 (0.03)
growth rate	—	8.19	0.32	0.03	0.78	6.25	1.83	0.68	0.06	0.06

**Figure 3.**
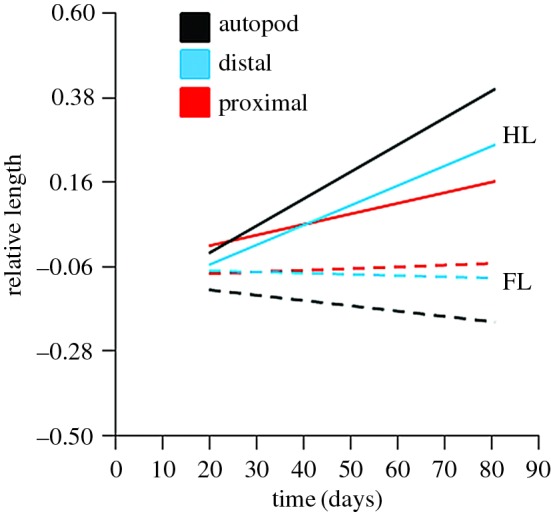
A regression analysis of the relative length of limb elements (proximal, distal and autopod) of *Pogona vitticeps* during embryogenesis (days post-oviposition), with the effects of allometry removed. Dashed and solid lines indicate forelimb elements (FL) and hindlimb elements (HL), respectively.

A regression analysis of the relative length of limb elements (proximal, distal and autopod), with the effects of allometry removed, found that there were significant differences between forelimb and hindlimbs during embryonic development (figure 3). A generalized linear model (GLM) incorporating the effect of LIMB*TIME found that there was a significant difference between forelimb and hindlimb during embryonic development for all limb elements (proximal: *F*_1,102_ = 6.86, *p* = 0.01; distal: *F*_1,102_ = 26.02, *p* < 0.001; autopod: *F*_1,102_ = 27.46, *p* < 0.001). A regression of each limb element individually, with the effects of allometry removed, found that the relative length of all hindlimb elements increased significantly during embryonic development (proximal: *r* = 0.51, *F*_1,51_ = 17.59, *p* < 0.001; distal: *r* = 0.67, *F*_1,51_ = 41.65, *p* < 0.001; autopod: *r* = 0.64, *F*_1,51_ = 35.64, *p* < 0.001), while there was no significant change in relative length of forelimb elements (proximal: *r* = 0. 10, *F*_1,51_ = 0.55, *p* = 0.46; distal: *r* = 0. 07, *F*_1,51_ = 0.25, *p* = 0.62; autopod: *r* = 0. 17, *F*_1,51_ = 1.53, *p* = 0.22). Regarding the length of hindlimb elements in the first time period for which separate elements could be measured (20 days post-oviposition), relative proximal length was greater than that of both distal and autopod limb elements (figure 3). Then, during late morphogenesis and throughout the growth phase, the relative lengths of distal and autopod elements exceeded that of the proximal hindlimb element. In hatchling lizards, the autopod is relatively longer than the distal and then proximal hindlimb elements. A different pattern of relative growth was observed in forelimbs (figure 3), where relative length of the proximal element remained fairly constant throughout development, while the relative lengths of distal and autopod elements decreased.

We calculated the average percentage growth rate per day from one sampling period to the next (table 1) and found that growth rates in both forelimb and hindlimbs were greatest in the 25 d (embryonic stage 36) to 28–29 d (embryonic stage 37) post-oviposition period, which corresponds to the transition from the morphogenesis to the growth phases of embryonic development. We used a *t*-test to compare growth rates to a null hypothesis (H_0_) of constant growth (forelimb = 13.2%/day; hindlimb = 19.3%/day). For hindlimb, we could significantly reject the H_0_ of a constant growth rate, indicating that growth rates vary significantly during limb development (*t* = −2.44; d.f. = 8; *p* = 0.04), with the 28–29 d post-oviposition (embryonic stage 37) period being a significant outlier (studentized residual = 6.67). We were unable to reject the H_0_ of a constant growth rate in forelimbs (*t* = −1.15; d.f. = 8; *p* = 0.28); however, the 28–29 d post-oviposition (embryonic stage 37) period was identified as a significant outlier (studentized residual = 3.15).

### 2.3. Expression of *Pitx1, Shh* and *GHR* in *Pogona vitticeps*

**Figure 4.**
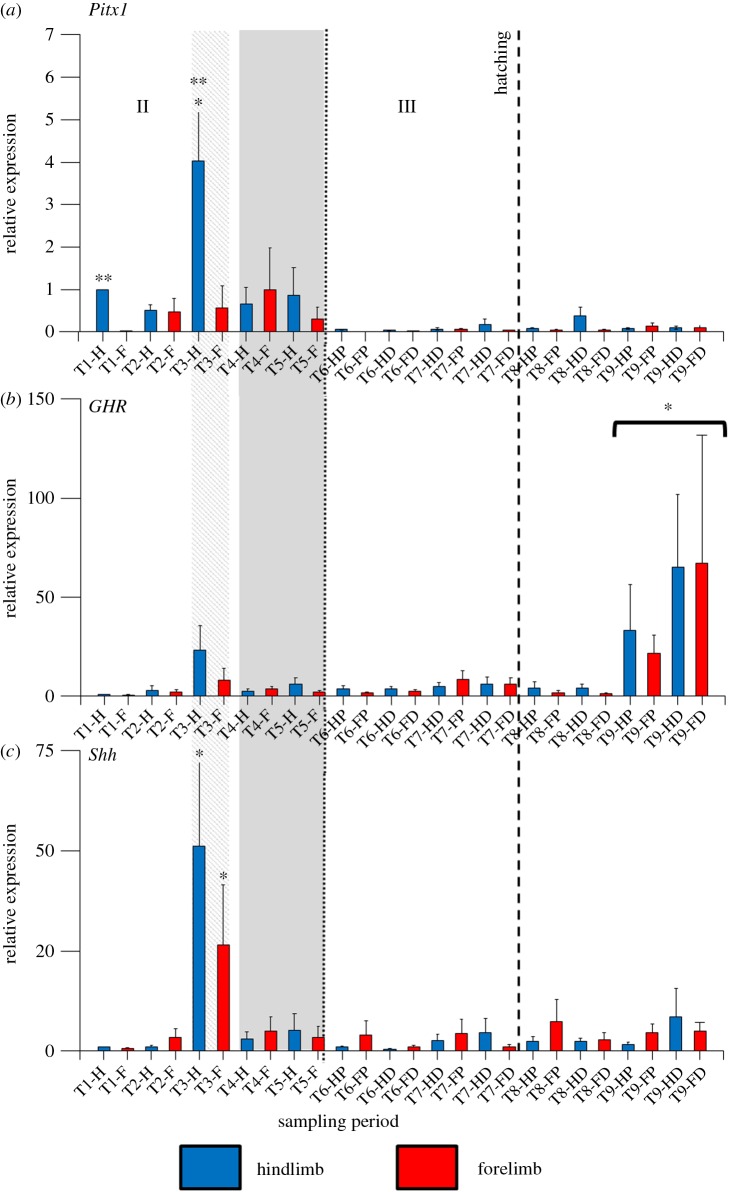
Transcript levels of three genes (*Pitx1, GHR, SHH*) in limb elements during development in *Pogona vitticeps.* Shown are results of quantitative reverse transcription PCR. Expression is relative to the earliest hindlimb time point (T1; 12–13 days post-oviposition). Development stages (T1–T9) on *y*-axis are detailed in table 1, with samples designated as forelimbs (F) or hindlimbs (H) and proximal (P) or distal elements (D) in later-stage embryos and hatchlings. Stages of embryogenesis are indicated: II, morphogenesis; III, growth. Stippled vertical bar indicates when forelimb and hindlimb lengths were found not to differ significantly and both were shorter than head length, while the shaded vertical bar shows the period of embryogenesis when differentiation between forelimb and hindlimb lengths occurs, with hindlimb length becoming significantly longer than forelimb length. One asterisk indicates significantly (*p* < 0.05) higher transcript levels in the same limb-type (i.e. forelimb or hindlimb) when compared with other developmental stages; two asterisks indicate significantly (*p* < 0.05) higher transcript levels between forelimb and hindlimb elements at the same developmental stage.

**Figure 5.**
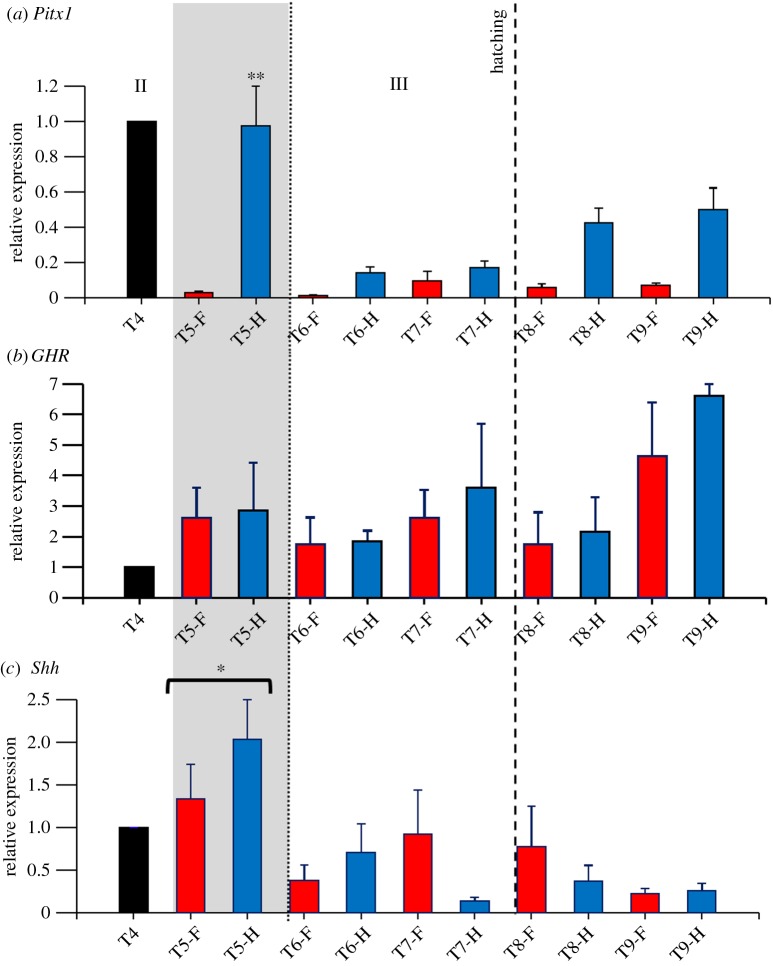
(*a*–*c*) Transcript levels of three genes (*Pitx1, GHR, SHH*) in autopods (hands/feet) during limb development in *Pogona vitticeps.* Shown are results of quantitative reverse transcription PCR. Transcript levels are relative to those in the earliest hindlimb autopod time point investigated (T4–25 days post-oviposition). Development stages (T4-T9) on *y*-axis are detailed in table 1, with samples designated as forelimb (F) or hindlimb (H) autopods. Stages of embryogenesis are indicated: II, morphogenesis; III, growth. Shaded vertical bar shows the period of embryogenesis when differentiation between forelimb and hindlimb lengths occurs, with hindlimb length becoming significantly longer than forelimb length. One asterisk indicates significantly (*p* < 0.05) higher expression in the same limb-type (i.e. forelimb or hindlimb) when compared with other developmental stages; two asterisks indicate significantly (*p* < 0.05) higher expression between forelimb and hindlimb elements at the same developmental stage.

In *P. vitticeps*, all three genes are transcribed in all parts of the limb at each of the stages of limb development examined, and transcript levels relative to those at the earliest hindlimb time point (12–13 days post-oviposition) were quantified (figures 4 and 5).

*Pitx1* showed highest transcript levels during morphogenesis in all hindlimb elements (figures 4 and 5) and in the distal and proximal sections of the forelimb (figure 4), while transcript levels in the forelimb autopods (figure 5) were low. A two-way ANOVA for the results depicted in figure 4 (whole limb buds in early embryogenesis and proximal and distal segments in later stages) found that there was a significant difference in transcript levels of *Pitx1* between time periods (*F*_8,135_ = 6.84; *p* < 0.001), and between forelimb and hindlimb elements (*F*_1,135_ = 11.74; *p* = 0.001). In addition, we found a significant interaction in transcript levels between limb type and time period (*F*_8,135_ = 4.19; *p* < 0.001). A Tukey's post hoc pairwise comparison test revealed a significant difference in transcript levels of *Pitx1* between forelimb and hindlimbs at 12–13 d (qPCR time period T1; *p* < 0.001) and 20 d (qPCR time period T3; *p* = 0.04) post-oviposition, while in the hindlimb *Pitx1* was transcribed at a significantly higher level at 20 d post-oviposition (qPCR time period T3) than at any other time period (*p* ≤ 0.002). Similarly, a two-way ANOVA for the results depicted in figure 5 (autopods) showed a significant difference in *Pitx1* transcript levels between time periods (*F*_4,50_ = 4.12; *p* = 0.006) and between forelimb and hindlimb elements (*F*_1,50_ = 28.87; *p* < 0.001), as well as a significant correlation between limb type and time period (*F*_4,50_ = 4.54; *p* = 0.003). A Tukey's post-hoc pairwise comparison test indicated a significant difference in *Pitx1* transcript levels between forelimb and hindlimbs at 15–18 d post-oviposition (qPCR time period T2; *p* < 0.001). These results indicate that highest levels of *Pitx1* transcription was in the hindlimbs at 20 d post-oviposition (qPCR time period T3), while in other time periods there was not a significant difference in *Pitx1 t* between forelimb and hindlimbs. In the growth phase of embryogenesis and after hatching, little to nil transcription was detected.

A two-way ANOVA for the results shown in figure 4 (whole limb buds in early embryogenesis and proximal and distal segments in later stages) found that there was a significant difference between transcript levels of *GHR* between time periods (*F*_8,135_ = 3.30; *p* = 0.002), but no significant difference between forelimbs and hindlimbs, and no correlation between limb type and time period. A Tukey's post hoc pairwise comparison test indicated a significantly higher level of *GHR* transcripts in 21 d hatchlings compared with that in any other time period (qPCR time period T9; *p* ≤ 0.02). A two-way ANOVA for the results depicted in figure 5 (autopods) found no significant difference in transcript levels of *GHR* between time periods or limb type. These results indicate that *GHR* showed highest transcript levels after hatching in forelimb and hindlimb elements (qPCR time period T9; figure 4), with a similar trend of increasing transcript levels until after hatching in the autopods (figure 5).

A two-way ANOVA for the results shown in figure 4 (whole limb buds in early embryogenesis and proximal and distal segments in later stages) found that there was a significant difference between transcript levels of *Shh* between time periods (*F*_8,135_ = 10.73; *p* < 0.001), but no significant difference between forelimb and hindlimbs, and no correlation between limb type and time period. A Tukey's post hoc pairwise comparison test indicated that there was a significant higher level of *Shh* transcript levels in 20 d post-oviposition (qPCR time period T3) embryos compared with any other time period (*p* < 0.001). Similarly, a two-way ANOVA for the results shown in figure 5 (autopods) found revealed a significant difference in transcript levels of *Shh* between time periods (*F*_4,50_ = 5.44; *p* = 0.001), but not between forelimb and hindlimb autopods, and no correlation between limb type and time period. A Tukey's post hoc pairwise comparison test indicated that there was a significant higher level of *Shh* transcript levels in the autopods of 28–29 d post-oviposition (qPCR time period T5) embryos compared with later time periods (*p* ≤ 0.01). These results indicate that *Shh* transcript levels peaked at 20 d post-oviposition during morphogenesis in both forelimb and hindlimb elements (figure 4), and at 28–29 d post-oviposition in hindlimb autopods (figure 5).

### 2.4. Immunohistochemistry: *Pitx1* expression in *Pogona vitticeps*

**Figure 6.**
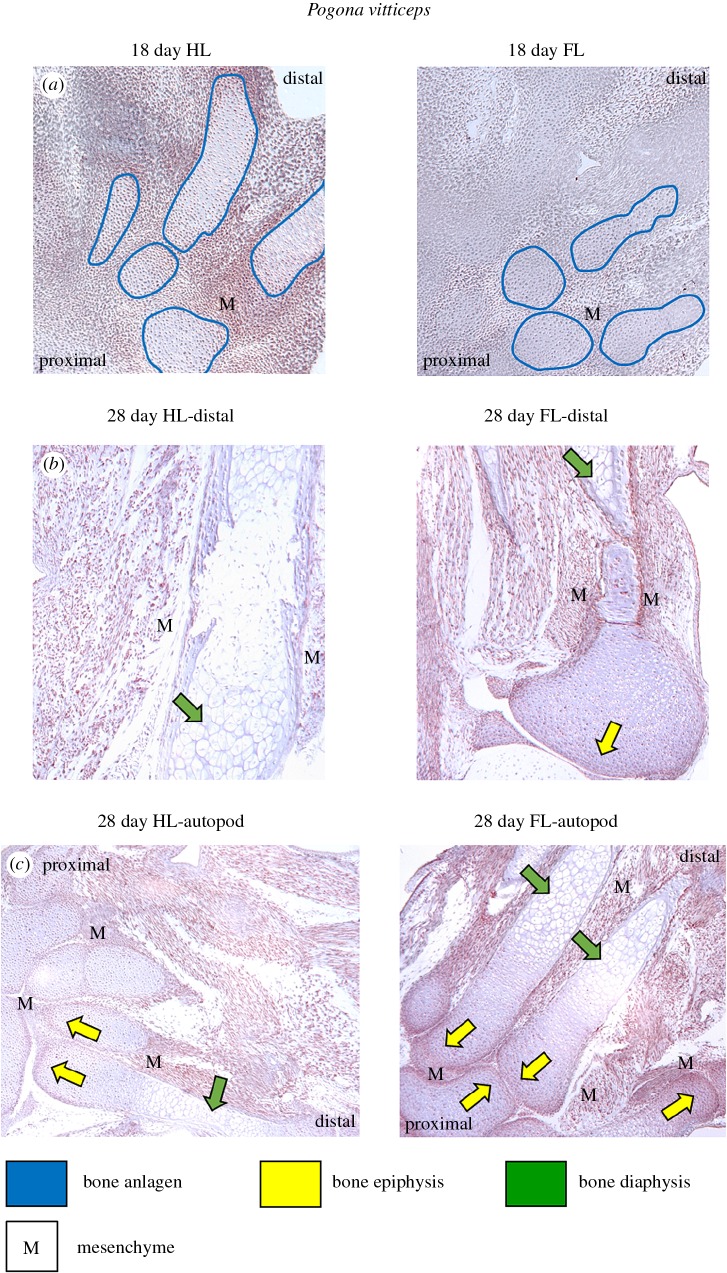
Detection of *Pitx1* expression in embryonic limb tissue of *Pogona vitticeps,* using immunohistochemistry (IHC)*.* (*a*) Eighteen-day post-oviposition forelimbs (FL) and hindlimbs (HL), showing the future autopod region (equivalent position to (*c*)), with formation of bones at base of the fingers/toes. The anlagen of the future bones is designated with blue lines, with strong, brown DAB staining in the mesenchyme around the condensation and more limited staining in the mesenchymal condensation (details of these areas are provided in figure 7). (*b*) Twenty-eight-day post-oviposition sections of the mid-distal section of forelimbs (FL) and hindlimbs (HL), with strong DAB staining in the mesenchyme around the long bones. (*c*) Twenty-eight-day post-oviposition sections of the autopods for both forelimbs (FL) and hindlimbs (HL). Strong DAB staining in the mesenchyme (labelled as ‘M’) around the base of the digital bones, particularly along planes of cartilage condensation and at digital joint formation, with more limited staining in the growth plates of the bone epiphysis (yellow arrows) and absence of staining in the chondrocytes of the bone diaphysis (green arrows). Nuclei are stained with haematoxylin (pale blue). Images shown are at 10× magnification. Proximal and distal directions are provided on images to allow orientation. Additional and uncropped images are available in electronic supplementary material, figure S2.

**Figure 7.**
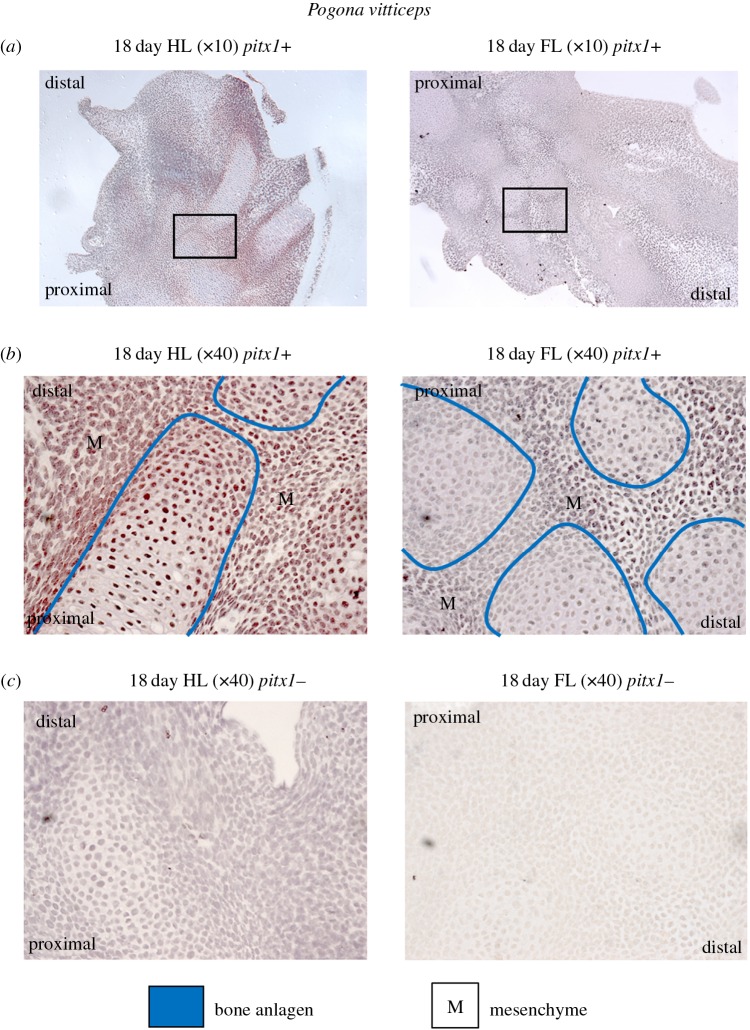
Detection of *Pitx1* expression in 18-day post-oviposition embryonic limb tissue of *Pogona vitticeps.* Shown are future autopod regions (equivalent position to figure 6*c*) for fore and hindlimb: (*a*) primary-antibody-positive sections (+) at magnification 10× with a location box for the magnification 40× images; (*b*) primary-antibody-positive sections (+) at magnification 40×; and (*c*) negative control sections (−) at magnification 40×. The anlagen of the future bones are designated with blue lines, with expression of *Pitx1* reflected by brown DAB staining, reminiscent of cell nuclei. Strong DAB staining in the mesenchyme (labelled as ‘M’) around the base of the digital bones, particularly along planes of cartilage condensation and at digital joint formation. Nuclei are stained with haematoxylin (pale blue). Proximal and distal directions are provided on images to allow orientation.

We detected PITX1 in both the hindlimb and forelimb sections of *P. vitticeps* (figures 6 and 7), which parallels *Pitx1* transcript levels (figure 4)*.* Using IHC, we applied 3-diaminobenzidine (DAB) as an easily detectable, brown chromogen (see ‘Material and methods’). At 18 days post-oviposition (qPCR time period T2), *Pitx1* is expressed in the forelimb and hindlimb sections (figures 6*a* and 7*a*,*b*). Expression in the forelimb is not as extensive or strong as in the hindlimb, but a comparison of the forelimb primary-antibody-positive sections with the negative control sections shows robust DAB staining (figure 7). DAB staining patterns are reminiscent of cell nuclei, probably indicating localization of the transcription factor PITX1. Expression in both the forelimbs and hindlimbs is concentrated in mesenchyme tissue, particularly along planes of cartilage condensation and at digital joint formation (figure 6*a*). Likewise, *Pitx1* is expressed in both hindlimbs and forelimbs 28 days post-oviposition (qPCR time period T5), with both distal segments and autopods showing expression concentrated in mesenchyme tissue (figure 6*b*,*c*). We also screened for *Pitx1* expression using IHC in forelimb and hindlimbs of 55-day post-oviposition (qPCR time period T7) embryos and 7-day hatchlings (qPCR time period T8). However, congruent with our results obtained with RT-qPCR (figure 4), in these subsequent developmental stages we did not detect *Pitx1* expression (data not shown).

The IHC results are consistent with our qPCR results with regard to the proximal and distal limb segments, where we observed a peak in *Pitx1* transcript levels during morphogenesis (figure 4), up until the 28–29-day post-oviposition stage (qPCR time period T5), followed by little discernible transcript levels during the growth phase and post-hatching. Conversely, in autopods we detected low levels of *Pitx1* transcripts in the forelimbs and hindlimbs of 55-day post-oviposition embryos (qPCR time period T7) and 7-day hatchlings (qPCR time period T8), and no expression was detected in our IHC screening in the autopods (results not shown). Thus, absolute transcript levels of *Pitx1* in the autopods at 55-day post-oviposition (qPCR time period T7) and 7-day hatchling (qPCR time period T8) stages may be very low and not give rise to a detectable amount of expressed PITX1.

### 2.5. *Pitx1* expression in *Ctenophorus pictus*

**Figure 8.**
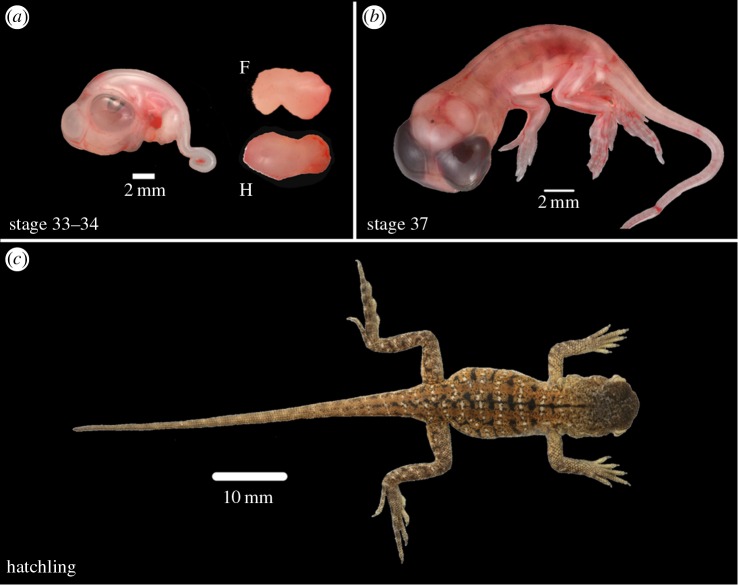
Embryonic stages in *Ctenophorus pictus*. Limb development stages included in our study for *C. pictus* include: (*a*) stage 33–34, morphogenesis, cartilaginous anlagen of the limb bones form (16 day post-oviposition); (*b*) stage 37, transition from morphogenesis to growth phase (30 day post-oviposition); and (*c*) hatchling (21 days post-hatching). Whole embryos/hatchlings are shown (scales bars provided) and for embryonic stage 33–34, images of forelimb (F) and hindlimb (H) are provided (not to scale).

Developmental stages of *C. pictus* embryos at 16 and 30 days post-oviposition (figure 8) were determined to be equivalent to stage 7 and 12–13 of the *Anolis* staging series [1], stages 31–32 and 37 of *L. vivipara* [31], and 12–13 days and 28–29 days post-oviposition in *P. vitticeps*, respectively (electronic supplementary material, S1). Limb measurements of these embryonic stages are provided in table 2.

**Table 2.** Morphological measurements for the three developmental stages of *Ctenophorus pictus*. Values presented are mean length (mm ± s.e.) taken from the right limb. Images provided in figure 8.

embryonic stage	limb element	*n*	forelimb	hindlimb
16 d post-oviposition	bud	4	2.3 ± 0.05	2.8 ± 0.05
30 d post-oviposition	proximal	4	2.5 ± 0.22	3.3 ± 0.23
	distal		2.0 ± 0.13	3.5 ± 0.21
	autopod		3.5 ± 0.38	6.6 ± 0.68
hatchling	proximal	6	5.3 ± 0.20	7.2 ± 0.26
	distal		4.0 ± 0.15	6.7 ± 0.46
	autopod		1.9 ± 0.12	3.1 ± 0.14
	fourth digit		4.4 ± 0.30	9.9 ± 0.32

**Figure 9.**
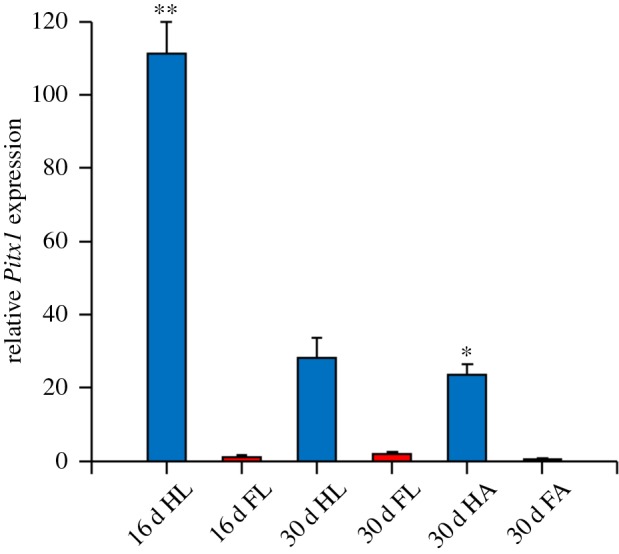
Expression of PITX1 in *Ctenophorus pictus*. Relative PITX1 transcript levels determined through qPCR in limb elements during development in *C. pictus*. Data points are mean ± s.e.m. All data are represented as relative to the amount of gene transcripts at 30-day post-oviposition in autopod forelimb (30 d FA) tissue. Forelimb and hindlimb tissue with significantly different levels of gene expression are indicated by *p < 0.01 and **p < 0.001.

**Figure 10.**
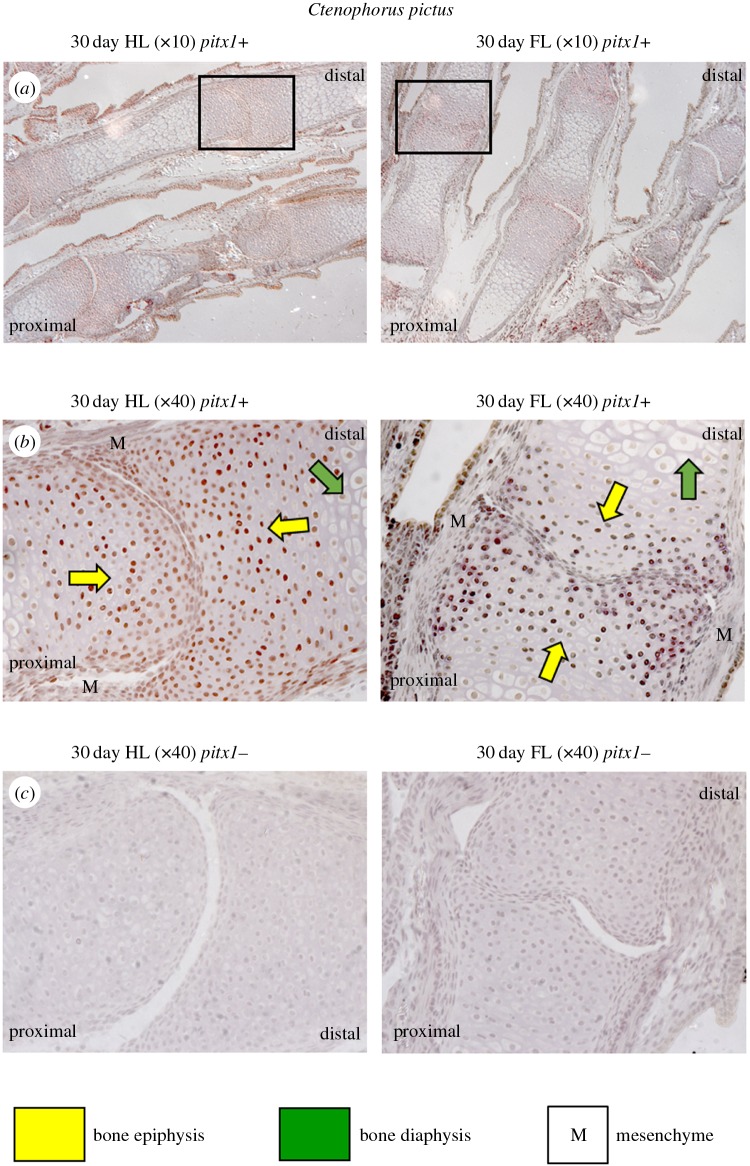
Detection of *Pitx1* expressed in embryonic limb tissue of *Ctenophorus pictus* at 30 days post-oviposition, using immunohistochemistry (IHC). Shown are autopods (equivalent position to figure 6*c*) for fore and hindlimb: (*a*) primary-antibody-positive sections (+) at magnification 10× with a location box for the magnification 40× images; (*b*) primary-antibody-positive sections (+) at magnification 40×; and (*c*) negative control sections (−) at magnification 40×. DAB staining in the mesenchyme (labelled as ‘M’) around the base of the digital bones, particularly along planes of cartilage condensation and at digital joint formation, also with staining in the growth plates of the bone epiphysis (yellow arrows) and the absence of staining in the chondrocytes of the bone diaphysis (green arrows). Nuclei are stained with haematoxylin (pale blue). Proximal and distal directions are provided on images to allow orientation.

*Ctenophorus pictus* shows strongest transcription levels of *Pitx1* (RT-PCR, 30 cycles) in the hindlimb (figure 9), while *P. vitticeps* expresses *Pitx1* (RT-PCR, 30 cycles) strongly in both the forelimbs and hindlimbs (electronic supplementary material, figure S3). Using qPCR, we found that there were high levels of *Pitx1* transcripts in the hindlimbs of *C. pictus* during morphogenesis, but low to nil expression in the forelimbs. Transcription was significantly higher in the hindlimb compared with the forelimb in the 16-day post-oviposition embryo (*t*2 = 11.7522, *p* = 0.007). Similarly, transcription was significantly higher in the hindlimb autopod at 30 days post-oviposition compared with the forelimb autopod (*t*3 = 21.89, *p* < 0.001). Similarly, in the 30-day post-oviposition hindlimb *Pitx1* transcript levels were higher than in the forelimb, but this was not significant (*t*2 = 2.5715, *p* = 0.124). IHC results are consistent with our qPCR results in *C. pictus* (figure 10). Unlike during late morphogenesis in *P. vitticeps* (figure 6), *C. pictus* was found to have significant differences in expression of *Pitx1* in forelimb and hindlimb sections. In the hindlimb sections of the autopods, PITX1 expression in *C. pictus* was similar to that in *P. vitticeps*, with strong DAB staining in the mesenchyme around the digital bones and developing claws, more limited staining in the growth plates of the bone epiphysis, and absence of staining in the chondrocytes of the bone diaphysis (figure 6).

## 3. Discussion

### 3.1. Limb development during embryogenesis in *Pogona vitticeps*

We identified eight morphological stages in *P. vitticeps,* corresponding to distinct developmental stages in *L. vivipara* [31] and *A. sagrei* [1]. In addition, we compared staging of *P. vitticeps* with that of agamid embryonic series, *Agama imparlearis* [32] and *Calotes versicolor* [33], although these studies illustrated development of forelimbs rather than hindlimbs. We found strong similarities between these published staging series and development in *P. vitticeps.* Additionally, in the oviparous *P. vitticeps*, we found that early embryogenesis occurs within the oviducts prior to oviposition, and eggs are laid at the limb-bud outgrowth and patterning stage of development (stage 28–30). It has been found that multiple-clutching oviparous squamates, such as *P. vitticeps,* retain eggs in their oviducts and lay them between embryonic stage 20 and 35 depending on the species [34].

Although we were unable to find a published embryonic staging series for *P. vitticeps*, there has been some comparative work relating embryonic development in this species to other squamates [35]. However, embryos were only examined up to stage 36. Our study, examining limb development past hatching, therefore provides an important foundation to comparative studies in limb development of agamid lizards. In *Anolis*, it has been shown that post-hatching growth trajectories for trunk-ground versus trunk-crown habitat specialists are consistently the result of changes that occur prior to hatching [7], and differences in limb length are apparent at hatching, with limb long bones elongating in parallel relative to body size in different species after hatching. As such, species-specific morphologies are the result of changes that occur very early in limb development, prior to formation of the cartilaginous anlagen [7]. We found that the greatest phase of embryonic limb growth in *P. vitticeps*, as a proportion over time, is during the transition from morphogenesis to the growth stage of limb development (stage 36–37) for both forelimbs and hindlimbs. We also found that onset of differentiation between forelimb and hindlimb length occurred at 20 days post-oviposition, which corresponds to stage 35. At this stage of development, we detected significantly elevated transcript levels of *Pitx1* and *Shh*. However, whether these pre-hatching patterns of limb development in *P. vitticeps* define the time point when interspecific variation in limb length arises in agamid lizards is yet to be determined. Thus, our study provides a foundation for future comparative work in Agamidae.

### 3.2. Gene expression

This study confirmed for the first time that key developmental genes are transcribed (*Pitx1*, *GHR* and *Shh*) and expressed (*Pitx1)* in limbs of the embryos and hatchlings of *P. vitticeps*, as would be expected based on previous studies on model organisms. In addition, we here documented expression of *Pitx1*, a hindlimb-specific transcription factor, in the forelimb during morphogenesis. Transcript levels of *Pitx1* in the forelimb of *P. vitticeps* were less abundant than in the hindlimb during early morphogenesis, based on qPCR, and transcript levels were generally less abundant in late morphogenesis but did not differ between the forelimbs and hindlimbs. Expression of *Pitx1* during early morphogenesis was concentrated around mesenchymal condensation, and during late morphogenesis in the mesenchyme around digital bones and developing claws, with more limited staining in the growth plates of the bone epiphysis. By contrast, we found significantly lower or no transcription and expression of *Pitx1* in the forelimb when compared with the hindlimb of the Australian agamid *C. pictus*, either in early- or late-stage morphogenesis.

*Pitx1* is structurally conserved between the chicken and mouse, showing 92% similarity [14], and plays a fundamental role in limb identity in these model organisms. Additionally, in mice, it has been demonstrated that *Pitx1* influences the patterning of different tissue types of the limb during morphogenesis [17], which is completed by stage E14.5. Recently, research on the role of *Pitx1* in limb development of the iguanid lizard *A. sagrei*, using a micromass culture system, found that transcript levels of *Pitx1* were maintained in micromasses derived from hindlimb cells compared with those from forelimb cells [8]. By contrast, we here report *Pitx1* expression by qPCR and IHC in the forelimb of *P. vitticeps* during morphogenesis. Expression of *Pitx1* in the forelimb has been seen in a whole-mount *in situ* hybridization of a chick [16]. However, expression was spatially restricted to a small area of the autopod and only found in very early stages. Additionally, *Pitx1* expression has been detected at later morphogenic stages in the forelimb of the frog *Eleutherodactylus coqui*, but not at early limb-bud stages [36]. Authors hypothesized that the forelimb expression of *Pitx1* was, in this case, a developmental process peculiar to an organism in which metamorphic and embryonic stages are modified [36], as this species bypasses the tadpole stage and develops limbs within its eggs. With the addition of our results, with *Pitx1* expression in the forelimbs during morphogenesis in *P. vitticeps*, we highlight the need for further research across a wider range of tetrapods needed to more fully understand evolutionary variation in molecular processes underlying limb morphology.

Although *Shh* and *GHR* transcript levels did not differ between forelimbs and hindlimbs, we did find that they varied at different stages during embryogenesis. *Shh* showed greatest levels of transcripts at 20 days post-oviposition in proximal and distal limb elements, which equates to morphogenesis (electronic supplementary material, S1). In this stage, the cartilaginous anlagen of the limb bones form the distal tips of digits that are free from digital webbing; digit 4 is notably longer than other digits, the limbs are flexed 90° caudally at elbows, and the digit joints are not yet obvious. This stage of development also corresponds with the period when forelimbs and hindlimbs start to differentiate in length (figure 2). *Shh* activity is required for the maintenance of growth and patterning of intermediate and distal limb structures. Mice that lack *Shh* have limbs [37,38], but they are reduced and the skeletal pattern is severely truncated. In the autopods of *P. vittceps, Shh* showed a peak of transcription at 28–29 days post-oviposition, which defines the transition from morphogenesis to growth phase and is characterized by significant elongation of all limb elements, including digits. It is known that *Shh* is involved in autopod patterning [22,39], by acting as both a morphogen as well as a mitogen [40,41]. However, digit identity and patterning has already occurred by 28–29 days post-oviposition. It is possible that *Shh* is playing a role in extension during this period of embryogenesis. *Shh* has been shown to be involved in limb muscle formation, particularly to regulate directional muscle cell migration in the distal limb elements [42].

In contrast with the other two genes examined, *GHR* transcripts were most abundant in the limbs of hatchling lizards, in proximal and distal segments. GH coordinates the growth of multiple target tissues during development, including skeletal muscle, and it is believed that *GHR* exerts specific and direct effects on skeletal muscles [26]. *GHR* is highly expressed in skeletal muscle, where it induces the synthesis of IGF1 [27]. Both GH and IGF1 are important regulators of longitudinal growth [28]. Our results suggest that post-hatching is an important stage in limb growth, especially in the more distal elements. A recent study on bone growth in the brown bat *Eptesicus fuscus* also indicated that post-natal development was an important stage for growth [43]. These results indicate that the processes that mediate limb growth could occur at a range of developmental stages and that despite developmental biology focusing on early development, in the case of the agamids, the molecular processes mediating hindlimb length could also occur post-hatching.

### 3.3. Conclusion

In conclusion, ours is the first study to demonstrate the transcription (*Pitx1*, *GHR* and *Shh)* and expression (*Pitx1*) of key limb developmental genes in the agamids. Our study analysed different limb regions across developmental stages and demonstrated that transcripts of the three genes are detectable in both distal and proximal limb regions as well as in the autopod across a wide time frame. Most studies on limb development focus on early embryogenesis. We extended our observations to include hatchlings and showed that *Pitx1, Shh* and, in particular, *GHR* continue to be transcribed, with their gene products presumably playing a role in the developing limbs of hatchling lizards. The significant increase in hindlimb length relative to body size between the 25 and 28/29-day post-oviposition stage is further evidence that these later stages of development (embryogenesis) are very important in terms of hindlimb developmental growth. Our results highlight the need for further research in a wide range of tetrapods to fully understand the role of key developmental genes in the evolution of morphological diversity, such as the role of *Pitx1* in hindlimb determination and morphology. Our study on *P. vitticeps* and *C. pictus,* combined with the recently published *P. vitticeps* genome [12], illustrates that the Australian agamids can be further used to examine gene expression within and between species, and thus identify mechanisms through which diversity in limb length and morphology could have evolved.

## 4. Material and methods

### 4.1. Laboratory animal care

*Pogona vitticeps* (6 females and 3 males) and *C. pictus* (10 males and 18 females) were collected at the start of the mating season (September) from the Murray Sunset National Park, Victoria (34°50′ S, 141°40′ E), to produce eggs and hatchlings for this study. Lizards were housed in appropriate laboratory conditions outlined by Uller *et al*. [44,45]. A light and heat/basking source was provided for each cage, including a 50 W lamp (8.5 h photoperiod) and UV lights (12 h photoperiod). Temperatures in each cage varied between 30°C and 40°C, which is consistent with spring/summer temperatures in the Murray Sunset National Park. Lizards were fed crickets ad libitum*,* dusted with calcium and vitamin supplements (Repcal, Los Gatos, California), three times per week. Lizards were sprayed daily with water, in addition, provided with a water dish, and a moistened sand patch was provided for egg laying. Cages were checked daily for eggs, which were then transferred to individual containers half-filled with moist autoclaved vermiculite (1 : 7 autoclaved water: vermiculite). Containers were placed in a Thermoline incubator (Coburg North, Victoria) at 28°C.

### 4.2. Embryogenesis and gene expression in *Pogona vitticeps*

#### 4.2.1. Collection of embryonic and hatchling limb tissues

Limbs were collected from developing embryos and hatchling lizards to examine gene expression during limb development. Embryos were killed using 0.01 ml of 11.1 mg ml^−1^ sodium pentobarbitone injected into the egg and hatchlings were killed with an IP injection of 0.1 ml of 32.5 mg ml^−1^ sodium pentobarbitone. All embryos and hatchlings were photographed with a digital 13-megapixel Canon camera (EOSD5) operated by a computerized system, using Digital Photo Professional. Stages of embryonic development were estimated using published embryonic staging in *L. vivipara* [31] and *A. sagrei* [1]. In addition, we compared staging of *P. vitticeps* with that of agamid *Ag. imparlearis* [32]. Limb length was measured from the digital photographs at each of the developmental stages to quantify limb growth during development, using ImageJ (v. 1.38, NIH, USA) to the nearest 0.001 cm. All statistical analyses were undertaken using SYSTAT v. 13 (Cranes Software International).

Tissues were collected differently for each developmental stage due to variation in limb size: (i) 12–20-day post-oviposition embryos—whole limb buds were removed in one piece; (ii) 25–29-day post-oviposition embryos—limbs were removed in two sections (autopods (foot/hand) and limbs); and (iii) 44-day post-oviposition embryos to hatchling lizards. Limbs were removed in three sections: proximal (femur/humerus), distal (tibia and fibula/radius and ulna) and autopods. Limbs were collected and placed immediately on dry ice, then stored in −80°C.

#### 4.2.2. RNA extraction and cDNA synthesis

RNA was extracted from frozen tissue samples (20–100 mg), homogenized in TRI Reagent (Ambion, Applied Biosystems, Scoresby, VIC) according to the manufacturer's instructions with the Wig-L-Bug crescent shaker (Densply Rinn), with minor modifications [46]. The resulting RNA pellets were washed in 75% ethanol, air dried and resuspended in RNASecure water (Ambion). RNA samples were then treated with DNAse I (Ambion) at 37°C for 20 min to remove residual genomic DNA. RNA concentrations were measured on the NanoDrop ND-1000 Spectrophotometer (NanoDrop Technologies, Wilmington, DE, USA; Biolab, Scoresby, VIC) with an A260 : A280 ratio of more than 1.9. The presence of 18S and 28S ribosomal RNA was confirmed by gel electrophoresis on 1.2% TBE agarose gels. First strand cDNA synthesis used 1 µg total RNA in 30 µl reactions, with Superscript III reverse transcriptase (200 U µl^−1^; Invitrogen) and oligo d(T) (50 µmol, Applied Biosystems) according to the manufacturer's instructions. Samples were incubated at 25°C for 10 min, 50°C for 50 min, 85°C for 5 min to terminate the reaction and then stored at −20°C.

#### 4.2.3. RT-PCR

We first established if *Pitx1*, *GHR* and *Shh* were transcribed in the autopods, forelimbs and hindlimbs of *P. vitticeps* embryos and hatchlings during development. Oligonucleotide primers (electronic supplementary material, table S4) for the target genes were designed from published chicken sequences (GenBank accession numbers: XM_414626.2, AB012236.1 and NM_204821.1). We sequenced the target genes for the study species and a range of other lizards (in the case of *Pitx1* and *SHH*); alignment files are available in the Figshare digital repository and can be accessed at https://figshare.com/s/9e497ba0790fcb481d0b. RT-PCR was performed on 44-day post-oviposition embryos (*n* = 2) and 7-day-old hatchlings (*n* = 2) in 20 µl reactions with GoTaq Flexi (Promega, Annandale, NSW), 25 mM MgCl_2,_100 ng µl^−1^ forward and reverse oligonucleotide primers (Sigma Aldrich, Castle Hills, NSW) and 1 µl cDNA. A negative control, using water instead of cDNA, was included in each RT-PCR. The RT-PCR programme used for all samples consisted of 80°C for 2 min, then 40 cycles of 95°C for 30 s, 55°C for 1 min and 72°C for 1 min, with a final extension step at 72°C for 10 min. PCR products (12 µl) were visualized using gel electrophoresis on 1.2% TBE agarose gels with 6 µl SYBR safe stain. Hyperladder IV (Bioline Pty Ltd., Eveleigh, NSW) was included on each gel to estimate size of RT-PCR products. Amplification of the correct gene was confirmed by sequence analysis. The DNA was purified using ExoSAP (GE Healthcare, Ryldamere, NSW), according to the manufacturer's instructions, and sequenced by Macrogen (Korea). A BLAST search (http://www.ncbi.nlm.nih.gov/blast/Blast.cgi) confirmed that the correct gene had been amplified.

#### 4.2.4. Real-time quantitative PCR (qPCR)

Quantification of *Pitx1*, *GHR* and *Shh* transcript levels in the forelimb and hindlimb of *P. vitticeps* embryos and hatchlings were measured at each of the developmental stages by qPCR. TaqMan labelled fluorogenic probes with a quencher dye TAMRA (6-carboxyl-tetramethyl-rhodamine) at the 3′end and a FAM (6-carboxy fluorescent) reporter dye at the 5′ end (BioSearch Technologies Inc, CA) and primers were designed from the sequenced PCR products above using RealTimeDesign software (BioSearch) (electronic supplementary material, table S4). Alignment files, including qPCR primers and probes, are available in the Figshare digital repository and can be accessed at https://figshare.com/s/9e497ba0790fcb481d0b, demonstrating the 100% specificity in the study species. Samples were analysed using the relative comparative *C*_T_ method according to the Stratagene MxPro Protocol (http://www.scribd.com/doc/60305516/84/Comparative-Quantitation-Data-Analysis). In relative quantification, the qPCR data are presented relative to another gene, often referred to as an internal control. All qPCR reactions were carried out in triplicate using 96-well optical reaction plates (Bio-Rad Laboratories, Gladesville, NSW) in 20 µl volumes consisting of 1 µl cDNA, 2× SensiMix dU (Quantace, Alexandria, NSW), 0.8 µl primers (20 uM) and 0.4 µl probe (20 uM) using the Mx3000P qPCR System from Stratagene (Agilent Technologies, Mulgrave, Victoria).

The qPCR experiment was designed to quantify gene expression between all limb elements and different embryonic stages in the hindlimb and forelimb in two separate experiments due to restrictions on plate size. Six individuals (*n* = 6) for each time stage were included in the experiments. Experiment 1: whole forelimb and hindlimb buds for post-oviposition 12–13 d, 15–18 d, 20 d and 25 d forelimbs; whole limb without autopod for 25 d hindlimbs and 28–29 d forelimbs and hindlimbs; separate forelimb and hindlimb proximal and distal sections (without autopods) for post-oviposition 44 d, 53–55 d embryos; separate forelimb and hindlimb proximal and distal sections (without autopods) for hatchlings 7 dH and 21 dH. Each plate contained the above listed series of hind and forelimb buds, proximal and distal sections. Experiment 2: autopods from forelimb (except 25 d) and hindlimbs of post-oviposition 28–29 d, 44 d, 53–55 d embryos and hatchlings 7 dH and 21 dH. Each plate contained the above listed series of hind and forelimb autopods. The genes of interest (GOI) and the endogenous reference gene or internal control (*r28S*) were assessed in separate qPCR plates and the *C*_T_ values of each gene of interest were normalized to r*28S C*_T_ values. The relative difference in expression was calculated using 12–13 d HL (earliest time point) for experiment 1 and 25 d (earliest time point) for experiment 2 as the calibrator. This means that the relative quantity of the calibrator is automatically defined as 1.0 on the graph. The formula used to calculate fold change is
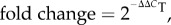
where ΔΔ*C*_T_ = [(*C*_T_ gene of interest − *C*_T_ internal control)] Sample A − [(*C*_T_ gene of interest − *C*_T_ internal control)] Sample B, where Sample A is the respective embryonic time point and Sample B is the calibrator. Standard deviations were calculated by taking the mean of qPCR replicates followed by the mean of the individual samples. Statistical tests and standard deviations were calculated after the 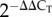 transformation, as described [47].

#### 4.2.5. Immunohistochemistry

To detect *Pitx1* expression in various stages of the developing embryonic limbs, the following time points were collected with *n = 5* per time point: 15–18-day post-oviposition forelimb and hindlimb buds; 28–29-day post-oviposition forelimb and hindlimb limbs and autopods; 53–55-day post-oviposition hind and forelimb proximal, distal and autopod sections; and 7-day hatchling hind and forelimb proximal, distal and autopod sections. The limbs and buds were embedded in paraffin, and transverse sections were cut and mounted on SuperFrost slides. Two sections from the same individual were mounted on the same slide, one for application of the primary antibody (*Pitx1*) and one as a negative control. Slides were de-waxed with a series of histolene, xylene and ethanol washes followed by antigen retrieval using Tris/EDTA/EGTA (pH9.0). A peroxidase block (30% hydrogen peroxide and 1×TBS) was used to block endogenous peroxidase for an hour followed by two 1×TBS washes. The sections on the slide were circled with a PAP pen (Abcam), which provides a hydrophobic barrier around the specimen. The sections were then blocked with a universal background Sniper (Biocare Medical) for 30 min with the excess blotted away. The primary polyclonal antibody Pitx1 (Bioworld Technologies) and a negative control (rabbit IgG) were added onto the sections at a 1 : 300 dilution (in PBS with BSA) each and incubated overnight at 4°C in a humidifying chamber. The secondary antibody, MACH4 Universal HRP (Biocare Medical), was added on the following day for 30 min after a series of TTBS washes. The slides were blotted and incubated with DAB (Vector Laboratories) for 2–10 min. Slides were viewed to check for brown colour development, then washed with distilled water. This was immediately followed by a DAB enhancer step, and then each slide was incubated for 10 min in 0.05 M sodium bicarbonate. The slides were then washed with MilliQ water, stained with haematoxylin, dehydrated through a series of ethanol and histolene washes, and then mounted in Cytoseal (ThemoScientific) and dried at 40°C overnight.

### 4.3. Pitx1 expression in *Ctenophorus pictus*

To investigate whether the pattern of *Pitx1* expression in *P. vitticeps* is typical across Australian agamid lizards, we selected a species, *C. pictus*, from a closely related genus of agamids [48].

We compared *Pitx1* transcription in the forelimbs and hindlimbs of *C. pictus* and *P. vitticeps* embryos using RT-PCR, which was undertaken at 16 days and 30 days post-oviposition for *C. pictus*, and 20 days, 29 days and 44 days post-oviposition for *P. vitticeps*, using the same protocol detailed above. In addition, the RT-PCR was repeated using 30 cycles to reduce the intensity of the PCR product.

We subsequently undertook qPCR of *Pitx1* at development stages of limb morphogenesis, one in early morphogenesis (16 days post-oviposition) and one in late morphogenesis (30 days post-oviposition), using the methods, primers and probes detailed above for *P. vitticeps*. All qPCR reactions were carried out in triplicate using 96-well optical reaction plates (Bio-Rad Laboratories, Gladesville, NSW). Four individuals (*n* = 4) for each time stage were included in the experiments. Samples were then analysed using the relative *C*_T_ standard curve method according to the Applied Biosystems User Bulletin #2 (http://www3.appliedbiosystems.com). A serial dilution, using distal hatchling limb tissue, was used to generate standard curves for *Pitx1* (50 ng µl^−1^, 10 ng µl^−1^, 5 ng µl^−1^, 1 ng µl^−1^, 0.5 ng µl^−1^) and the housekeeping gene (5 ng µl^−1^, 1 ng µl^−1^, 0.5 ng µ ^−1^, l 0.1 ng µl^−1^, 0.05 ng µl^−1^). The known initial concentration of RNA was plotted against the *C*_T_ value: the cycle at which the intensity of fluorescence, indicative of the amount of PCR product, crosses an arbitrary threshold, on a logarithmic scale. 33 ng µl^−1^ of cDNA was used for the analysis of gene of interest and 2 ng µl^−1^ for the housekeeping gene, *r28S*. All PCR reactions were carried out in triplicate using 96-well optical reaction plates (Bio-Rad Laboratories, Gladesville, NSW) in 20µl volumes consisting of 1 µl cDNA, 2× SensiMix dU (Quantace, Alexandria, NSW), 0.8 µl primers (20 µM) and 0.4 µl probe (20 uM) using the DNA Engine Opticon 2 System (MJ Research; Bio-Rad Laboratories). The experimental design to quantify differences in *Pitx1* expression between the forelimb and hindlimb contained tissue samples (16dHL, 16dFL, 30dHL, 30dFL, 30dHA and 30dFA) on one plate. *Pitx1* and the endogenous reference gene (*r28S*) were assessed in separate PCRs and the gene of interest *C*_T_ values were normalized to r*28S C*_T_ values. The relative difference in expression was calculated using 30HL as the calibrator.

IHC assays in *C. pictus* were undertaken at the time point (30 days post-oviposition), where there was least difference in *Pitx1* transcription between forelimbs and hindlimbs in *P. vitticeps*. We used the sample protocols in *C. pictus* as those in *P. vitticeps*, as detailed above, with *n = 4* individuals.

## Supplementary Material

Embryonic Staging in Pogona vitticeps

## Supplementary Material

Additional uncropped IHC images for Pogona vitticeps

## Supplementary Material

Transcription of Pitx1 in Ctenophorus pictus (CP) and Pogona vitticeps (PV) embryonic limbs

## Supplementary Material

Primers designed for this study, which were used in sequencing and in QPCR for the three limb development genes and ribosomal 28S in Pogona vitticeps

## Data accessibility

Primer and probe sequences, additional IHC results, and RT-PCR results can be found in the electronic supplementary material. The sequence alignment files supporting the results of this article are available in the Figshare digital repository and can be accessed at https://figshare.com/s/9e497ba0790fcb481d0b.
