# Supplementary material for: Expression of a hindlimb-determining factor Pitx1 in the forelimb of the lizard Pogona vitticeps during morphogenesis
Source: Open Biol. 2016 Oct 26;6(10):160252. doi: 10.1098/rsob.160252 (PMC5090065; doi:10.1098/rsob.160252)
Supplement: Embryonic Staging in Pogona vitticeps [file rsob160252supp1.pdf]

Appendix S1 Embryonic Staging in *Pogona vitticeps*

Stages of embryonic development were estimated using published embryonic staging in *Lacerta vivipara* (Dufaure and Hubert, '61) – a comparison to embryonic staging in *Anolis sagrei* (Sanger et al, '08) is provided in parentheses.

Late stage limb bud outgrowth and patterning - limb bud filled with undifferentiated mesenchyme.

Stage 31 (Anolis stage 6): nine days post-oviposition. Distal limb-bud paddle-shaped, which is distinct on both hindlimb and forelimb; forelimb flexes caudally.

Morphogenesis - cartilaginous anlagen of the limb bones form.

Stage 32 (Anolis stage 7): 12-13 days post-oviposition. Digit condensations first visible; first recognizable proximodistal segmentation of forelimb and hindlimb.

Stage 33 – 34 (Anolis stage 8): 15-18 days post-oviposition. Long bone condensations first visible; condensations of all digit cartilages visible; slight thinning, but no regression, of interdigital webbing; limb joints more distinct.

Stage 35 (Anolis stage 9): 20 days post-oviposition. Distal tips of digits freed of digital webbing; digit 4 noticeably longer than other digits; limbs flexed 90° caudally at elbows; digit joints not yet obvious.

Stage 36 (Anolis stage 11): 25 days post-oviposition Digital webbing mostly regressed, occasionally a remnant of webbing remains between digits 2 and 3; digit joints visible in digit 3 and 4.

Transition from morphogenesis to growth phase.

Stage 37 (Anolis stage 12-13): 28-29 days post-oviposition. Significant elongation of all limb elements, including digits; pinching at tip of digits delineating claws; all digit bones clearly visible; occasional scale papillae and pigmentation spots visible on hindlimbs; claws refractive to light along dorsal edge only.

Growth - mediated from epiphyseal growth zones.

Stage 38-39 (Anolis stage 17): 44 days post-oviposition. Fully developed scales on dorsal surface of limbs and scale anlagen on ventral surface; some overlapping scales on dorsal surface of limbs; clear calcification in long-bones; claws refractive to light; pigmentation patterns clearly visible on dorsal surface of limb; first visible striations on enlarged mucronate scales with dark pigmentation along the posterior edges.

Stage 40 (Anolis stage 19): 53-55 days post-oviposition. Fully formed animal still within egg. Limbs fully developed, claws pigmented; scales on limbs overlapping, pigmented and strongly keeled.

Hatching occurs when yolk fully consumed: 59-67 days post-oviposition.
