# Supplementary material for: Expression of a hindlimb-determining factor Pitx1 in the forelimb of the lizard Pogona vitticeps during morphogenesis
Source: Open Biol. 2016 Oct 26;6(10):160252. doi: 10.1098/rsob.160252 (PMC5090065; doi:10.1098/rsob.160252)
Supplement: Additional uncropped IHC images for Pogona vitticeps [file rsob160252supp2.pdf]

Appendix S2 Additional uncropped IHC images for *Pogona vitticeps*.

(All are raw, uncropped and unedited images at x10 magnification, taken with the same microscope settings).

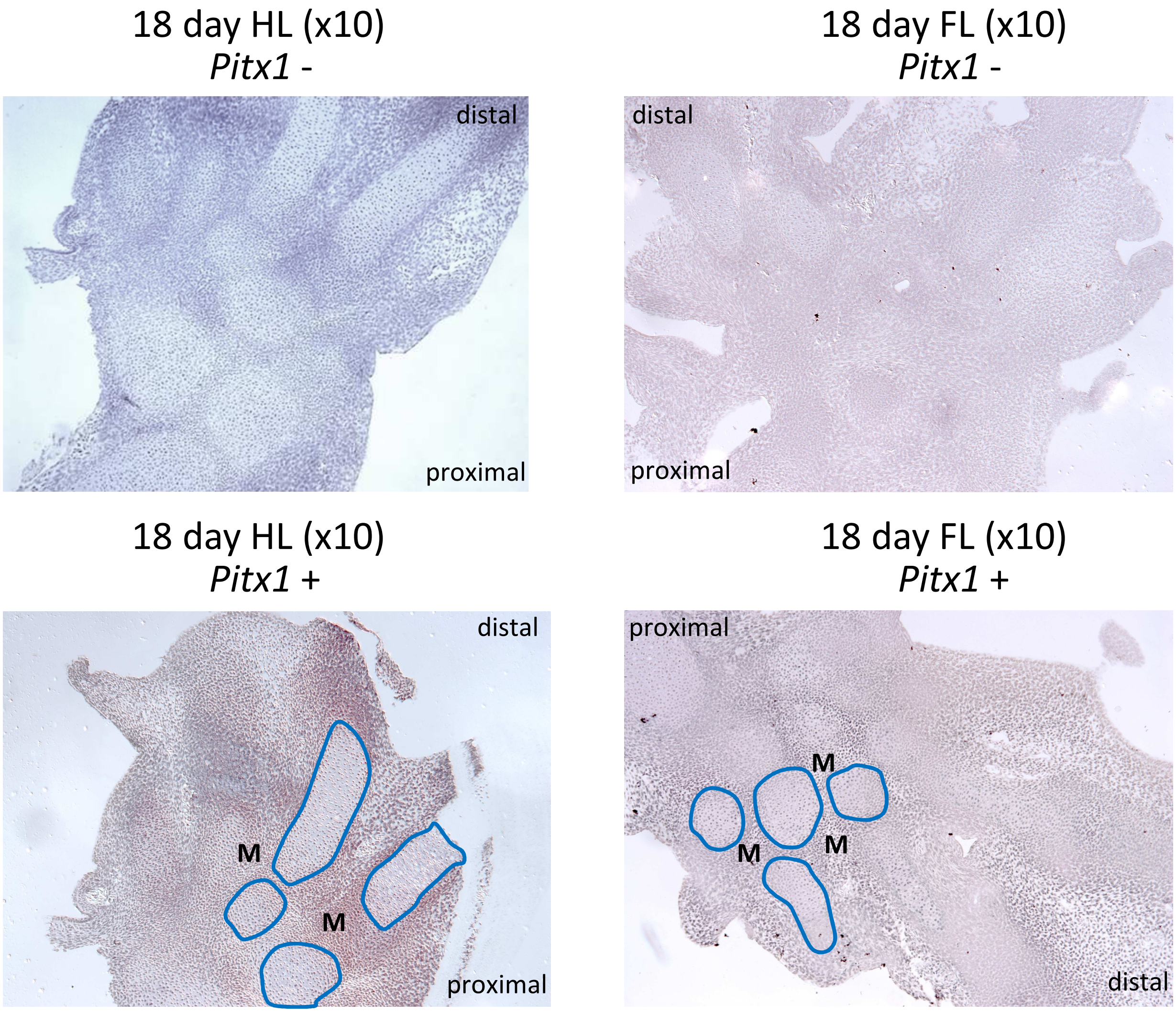

Figure S2a Detection of *Pitx1* expression in embryonic limb tissue of *Pogona vitticeps*, using immunohistochemistry (IHC).18 day post-oviposition forelimbs (FL) and hindlimbs (HL), showing the future autopod region (equivalent position to figure 6c), with formation of bones at base of the fingers/toes. Top row shows negative control sections (-) and bottom row shows primary-antibody-positive sections (+). For the *Pitx1* + sections, the anlagen of the future bones are designated with blue lines, with strong, brown DAB staining in the mesenchyme around the condensation and more limited staining in the mesenchymal condensation (details of these areas are provided in figure 7). Proximal and distal directions are provided on images to allow orientation.

bone anlagen | bone epiphysis | bone diaphysis

M mesenchyme

30 day HL (x10)
Pitx1 +
autopod

30 day FL (x10)
Pitx1 +
autopod

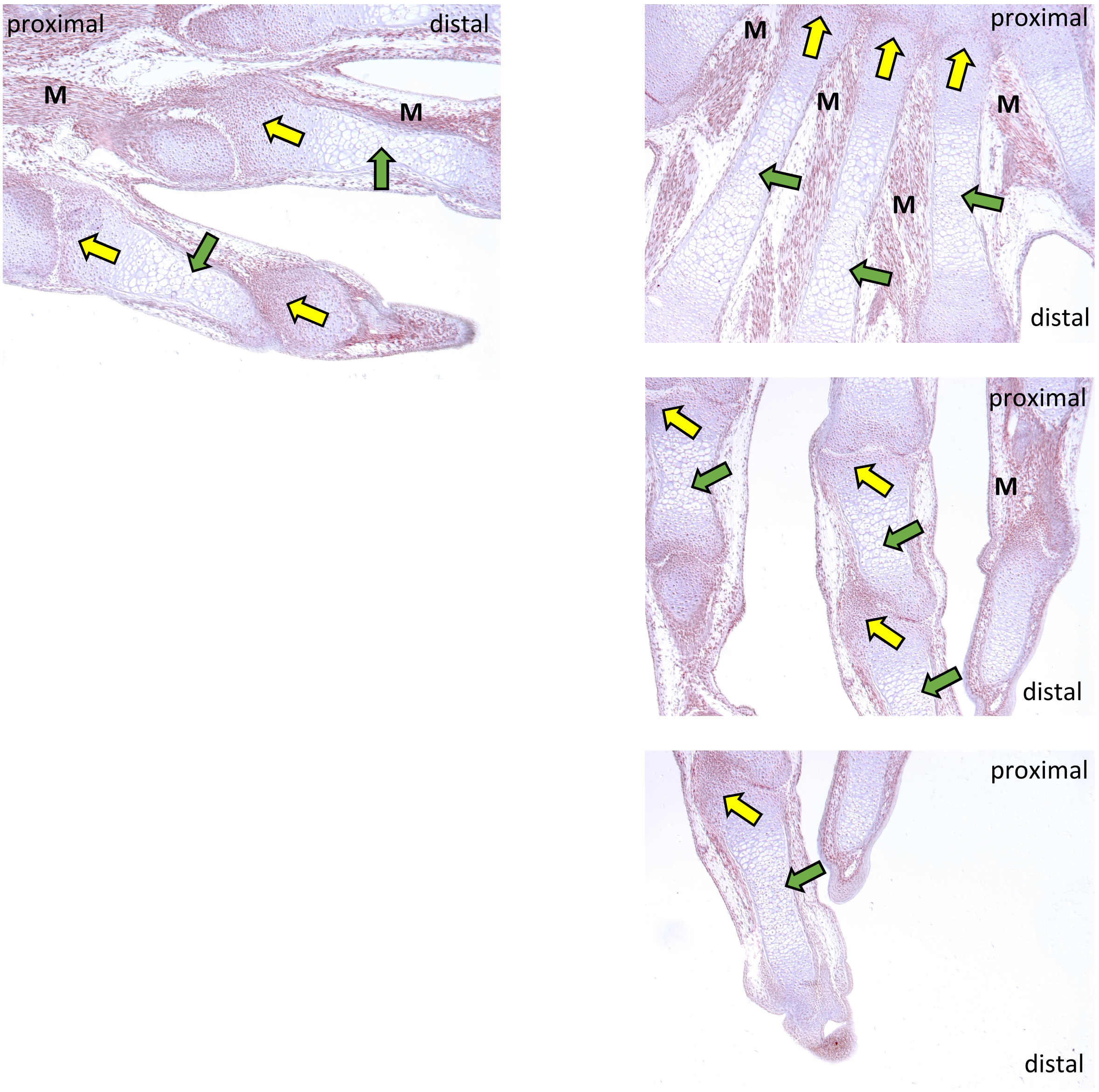

Figure S2b Detection of *Pitx1* expression in embryonic limb tissue of *Pogona vitticeps*, using immunohistochemistry (IHC). 30 day post- oviposition sections of the autopods for both forelimbs (FL) and hindlimbs (HL). Strong DAB staining in the mesenchyme (labelled as "M") around the base of the digital bones, particularly along planes of cartilage condensation and at digital joint formation, with more limited staining in the growth plates of the bone epiphysis (yellow arrows) and absence of staining in the chondrocytes of the bone diaphysis (green arrows). Nuclei are stained with hematoxylin (pale blue). Proximal and distal directions are provided on images to allow orientation.
