# Supplementary material for: Expression of a hindlimb-determining factor Pitx1 in the forelimb of the lizard Pogona vitticeps during morphogenesis
Source: Open Biol. 2016 Oct 26;6(10):160252. doi: 10.1098/rsob.160252 (PMC5090065; doi:10.1098/rsob.160252)
Supplement: Transcription of Pitx1 in Ctenophorus pictus (CP) and Pogona vitticeps (PV) embryonic limbs [file rsob160252supp3.pdf]

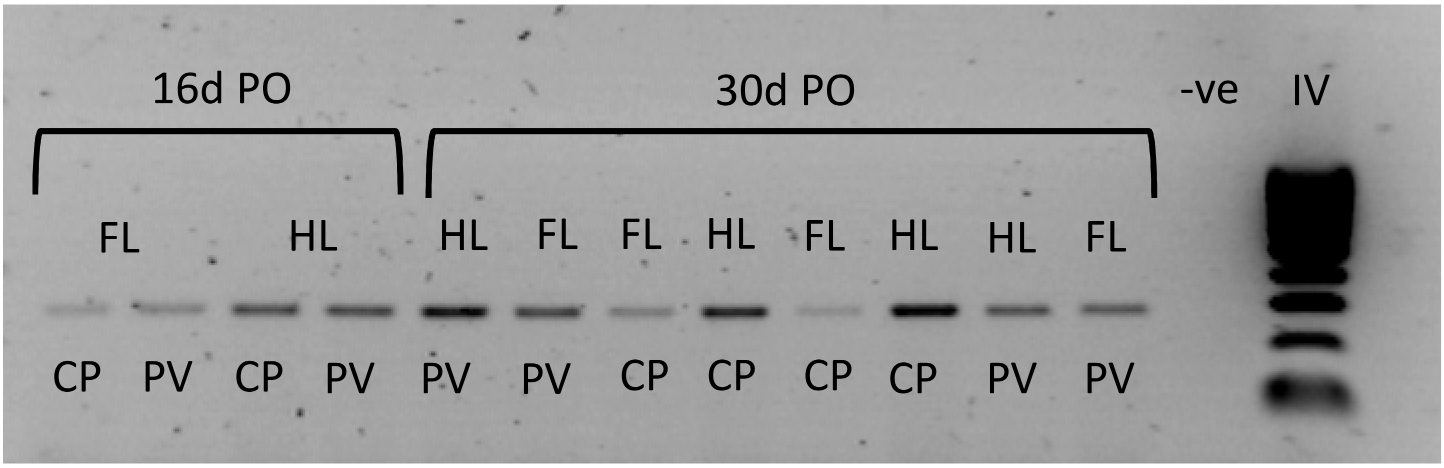

Figure S3. Transcription of *Pitx1* in *Ctenophorus pictus* (CP) and *Pogona vitticeps* (PV) embryonic limbs. Gel electrophoresis of PCR products after 30 cycles of denaturation, annealing and extension, with cDNA as template. Abbreviations are as follows: -ve= water negative control; IV =Hyperladder IV; PO = post-oviposition; HL = hindlimb; FL = forelimb.
