# Supplementary material for: Expression of a hindlimb-determining factor Pitx1 in the forelimb of the lizard Pogona vitticeps during morphogenesis
Source: Open Biol. 2016 Oct 26;6(10):160252. doi: 10.1098/rsob.160252 (PMC5090065; doi:10.1098/rsob.160252)
Supplement: Primers designed for this study, which were used in sequencing and in QPCR for the three limb development genes and ribosomal 28S in Pogona vitticeps [file rsob160252supp4.pdf]

Table S1. Primers designed for this study, which were used in sequencing and in QPCR for the three limb development genes and ribosomal 28S in *Pogona vitticeps*.

Primers and Probes	Sequence (5' to 3' direction)
<u>Sequencing Primers</u>	
Pitx1 F	TGGTTCAAGAACCGSCGAGCCAAGTGG
Pitx1 R	GTGTCCCGGTAVACSSTGTAGGG
Shh F	ATCTCGGTGATGAACCAGTGGCC
Shh R	TACCCAGTCGAAGCCGGCCTC
GHR F	CCATGGACARTGCMTATTTC
GHR R	GCTGGTCTGTGCTCACRTA
r28S F	GCAGGAGGTGTCAGAAAAGTTACC
r28S R	ACGCTTGGTGAATTCTGCTT
<u>QPCR primers and probes</u>	
Pitx1 F	CCAGTTCAGCGGCCTGATG
Pitx1 R	CGGGCTCATGGAGTTCAAGAAG
Pitx1 Probe	AGCCCTACGAGGACGTCTACGCG
Shh F	CGGAGGAGTCGCTTCACTAC
Shh R	CGGGCCAGCATCCCATAC
Shh Probe	AAGGCCGGGCTGTGGATATCACC
GHR F	GCCAGAAGTTCAAGAACAGAGCAT
GHR R	TGCGGTCGCAGGATTCAC
GHR Probe	TCTGAGGATGCTTACTTTGCCACCG
r28S F	CACCAGGGATAACTGGCTTGT
r28S R	CGCTTGGTGAATTCTGCTTCA
r28S Probe	CGGCCAAGCGTTCATAGCGACG
